# Evolution of dominance mechanisms at a butterfly mimicry supergene

**DOI:** 10.1038/ncomms6644

**Published:** 2014-11-27

**Authors:** Yann Le Poul, Annabel Whibley, Mathieu Chouteau, Florence Prunier, Violaine Llaurens, Mathieu Joron

**Affiliations:** 1 CNRS UMR 7205, Muséum National d’Histoire Naturelle, CP50, 45 rue Buffon, Paris 75005, France

## Abstract

Genetic dominance in polymorphic loci may respond to selection; however, the evolution of dominance in complex traits remains a puzzle. We analyse dominance at a wing-patterning supergene controlling local mimicry polymorphism in the butterfly *Heliconius numata*. Supergene alleles are associated with chromosomal inversion polymorphism, defining ancestral versus derived alleles. Using controlled crosses and the new procedure, Colour Pattern Modelling, allowing whole-wing pattern comparisons, we estimate dominance coefficients between alleles. Here we show strict dominance in sympatry favouring mimicry and inconsistent dominance throughout the wing between alleles from distant populations. Furthermore, dominance among derived alleles is uncoordinated across wing-pattern elements, producing mosaic heterozygous patterns determined by a hierarchy in colour expression. By contrast, heterozygotes with an ancestral allele show complete, coordinated dominance of the derived allele, independently of colours. Therefore, distinct dominance mechanisms have evolved in association with supergene inversions, in response to strong selection on mimicry polymorphism.

## Abstract (web summary)

The evolution of genetic dominance in polymorphic traits remains poorly understood. Here, the authors show that distinct dominance mechanisms have evolved in association with supergene inversions controlling wing pattern in *Heliconius* butterflies, in response to strong selection favouring mimicry.

## Graphical abstract

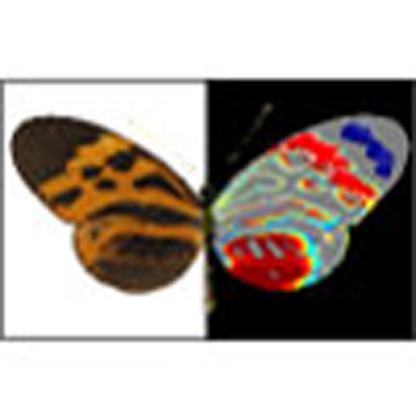

Dominance describes how the phenotype of heterozygous genotypes at a locus is determined by the relative expression of each allele. The mechanisms underlying variation in dominance have been strongly debated^1^. Dominance differences are often the direct effects of expression or activity levels of alleles, with dominant alleles being active and recessive ones having low expression or activity^2^. However, dominance variations are sometimes controlled by dominance modifiers such as *trans*-acting factors^3^. Dominance may respond to selection for traits showing high levels of local polymorphism, where a large proportion of individuals can be heterozygous^4^. Selection on dominance is also important during the spread of new alleles, which initially occur mostly at heterozygous state. This effect, called, ‘Haldane’s sieve’^5^,^6^,^7^, predicts that positive selection is more efficient when the new adaptive allele is dominant.

Loci showing adaptive polymorphism for complex traits are sometimes organized into supergenes with several tightly linked genes^8^ such as the locus controlling Batesian mimicry in butterflies^9^,^10^,^11^, the ‘social supergene’ in fire ants^12^ or the supergene controlling plumage and behavioural variation in the white-throated sparrow^13^. Recombination suppression in supergenes means that supergene alleles segregate as for a single Mendelian locus, composed of several component genes controlling different co-adapted characters^8^,^14^. However, both the genetic and evolutionary mechanisms by which dominance can be coordinated in such polygenic architectures remain unclear.

Supergenes controlling polymorphic mimicry are excellent cases to study the evolution of dominance coordination in complex traits because the fitness landscape underlying the evolution of warning colours can be easily predicted. Locally abundant warning signals are readily avoided by predators^15^,^16^ and characterize sharp peaks of fitness. Forms that are rare, and therefore unknown to local predators, define valleys of low fitness separating these peaks. In polymorphic populations, theory predicts selection against non-mimetic intermediate forms. In sympatry, this selection should favour coordinated dominance across the different pattern elements of the wings between supergene alleles. This does not hold between alleles found only in different populations and which are rarely brought together in heterozygotes^17^,^18^. This prediction was verified at a large geographic scale in the polymorphic species *Papilio dardanus*, engaged in Batesian mimicry with several distinct distasteful models^19^. In the single locus controlling polymorphic mimicry in *P. dardanus*^10^,^20^, dominance is stronger in crosses within a geographical race compared with inter-racial crosses^21^. Likewise, in hybrid zones separating two monomorphic populations of *Heliconius erato*, co-dominant alleles appear to be under stronger predation pressure than dominant alleles^22^,^23^. Here we study the evolution of dominance coordination at a polymorphic supergene controlling multimodal wing pattern variation involved in mimicry, where the supergene is well characterized at the molecular level, allowing investigating the evolution of the dominance mechanism at the supergene.

Neotropical butterflies in the genus *Heliconius* are famous examples of unpalatable prey engaged in Müllerian mimicry with other local butterflies. While the majority of *Heliconius* species are locally monomorphic and vary geographically^24^, *H. numata* maintains a high local polymorphism throughout its geographical range. These different forms are controlled by alleles of a single supergene locus called *P*^25^. Dominance among the different alleles of the supergene lies in a hierarchical series and is presumed to favour the mimicry of heterozygous individuals. However, the evolutionary mechanisms underlying the formation of this dominance series remain unclear.

At the *P* supergene, polymorphic inversions suppress recombination and result in complete linkage disequilibrium across a chromosomal segment containing at least 18 genes, maintaining the favourable genetic combinations that produce mimetic colour patterns^11^. These inversions distinguish two major allelic classes. A first class of alleles displays the ancestral gene order, common to butterflies and moths such as *H. melpomene* or *Bombyx mori*^11^,^26^, and is referred to as the ancestral class. Other alleles display rearranged gene orders^11^, but all share a 400-kb chromosomal inversion constituting a class of derived alleles. Ancestral alleles are generally recessive to the derived alleles but the functional mechanisms underlying dominance within and between allelic classes are unknown.

Butterfly wing patterns are formed by scales of different types each expressing a single colour. In several species, patterns are known to be determined by morphogen diffusion, with concentration gradients triggering the expression of different pigments across wing regions^27^. Dominance among patterning alleles could derive from the properties of colour pattern developmental pathways (for example, level of morphogen expression, rates of diffusion or transport through wing cells, or response thresholds)^28^. In the genus *Heliconius*, wing patterns are formed by scales with contrasting colours such as black, orange, red, yellow or white^29^, and the dominance relationships associated with the expression of those colours is often similar in different species^30^. However, variations in this hierarchy of colour expression exist, such as yellow and white patches being recessive in *H. melpomene* and *H. erato* but partly dominant in *H. cydno*^31^. This highlights the importance of this hierarchy in the evolution of dominance.

To investigate how selection has shaped dominance mechanisms between the multiple mimicry alleles of the supergene *P* in *H. numata*, we compared the phenotypes of heterozygotes from controlled crosses performed within and between populations. In this species, each population harbours a certain combination of mimetic alleles coexisting in sympatry and matching the local mimicry rings^32^,^33^. Mimetic patterns vary in complex combinations of size, colour and position of patches and boundaries across the wing. The quantification of dominance for the entire wing phenotype requires a method to encompass this complexity for comparisons between the wing patterns of heterozygotes relative to homozygotes, without postulating which part of the pattern variation needs to be measured. Our new procedure, Colour Pattern Modelling (CPM), quantifies variations in patch boundaries by modelling the distribution of the main colours through colour categorization. CPM defines homology between pattern positions across specimens through the recursive alignment of images to an average ‘model’ pattern, used as a reference.

By quantifying wing-pattern phenotypes with CPM, we find strict dominance in sympatry and inconsistent dominance throughout the wing between alleles from distant populations. We identify two mechanisms underlying dominance, associated with chromosomal inversions, suggesting an evolution of dominance mechanisms at the mimicry supergene.

## Results

### Overall dominance of wing patterns and local adaptation

Mimicry should favour strict dominance relationships between sympatric alleles (dominance coefficient close to 1), or else resemblance to another mimicry ring distinct from both homozygotes. On the other hand, dominance levels between parapatric alleles are not under selection and could thus take any value between 0.5 and 1.

**Figure 1:**
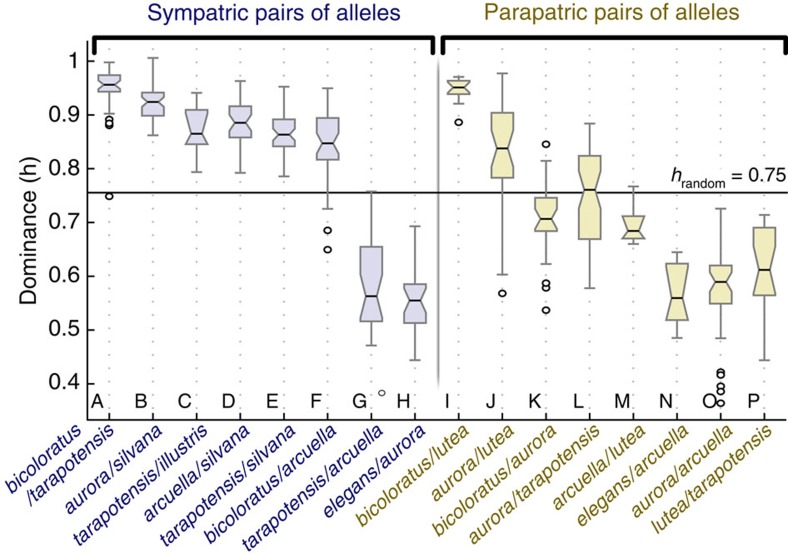
Variation of dominance between different pairs of supergene alleles. Box plot with the median, first, third interquartile range, extreme value and outliers represent the variation of dominance between pairs of alleles found in sympatry (blue) or parapatry (yellow). Dominance is measured in the direction of the most dominant allele (*h*=1: complete dominance; *h=*0.5: co-dominance).

To test whether selection has influenced dominance, we quantified dominance for sixteen sympatric and parapatric pairs of alleles (Fig. 1). Our estimates range from near complete dominance (*h*=0.95) to co-dominance (*h*=0.55). In accordance with expectation, dominance between pairs of sympatric alleles (*h*_mean_=0.83) was significantly stronger than between parapatric alleles (*h*_mean_=0.71; analysis of variance (ANOVA), *N*=472, F_1,470_*=*80,65, *P*<0.0001; significance was confirmed by permutation tests, see Supplementary Fig. 1).

Two pairs of sympatric alleles, *tarapotensis/arcuella* (Fig. 1, genotype G) and *elegans/aurora* (Fig. 1, genotype H) had low *h* estimates (*h* ~ 0.5), indicating overall co-dominance despite being sympatric. The *elegans* and *aurora* forms resemble each other, and, although they are controlled by distinct functional alleles segregating in crosses^11^,^25^, their phenotypic distance is the lowest of all pairwise comparisons so that the heterozygote may be similar enough to the homozygotes to benefit from mimicry protection. Indeed, we found that dominance between all pairs of alleles was positively correlated with the phenotypic distance between the two homozygotes (Pearson correlation, *N*=472, *R*^2^=0.45, *P*=0.0042), indicating that dominance was weaker when the respective homozygotes are phenotypically similar.

The phenotype of *tarapotensis/arcuella* heterozygotes, however, is clearly distinct from the *tarapotensis* or *arcuella* forms. These heterozygotes display a known morph referred to as *timaeus*, which belongs to another local mimetic ring dominated by Ithomiines (such as *Melinaea menophilus hicetas* or *Athyrtis mechanitis*)^32^,^34^. This is one of the well-represented local wing patterns in our study area^33^ and is also predominant in other parts of the Amazon basin where it attracts an even larger number of co-mimics, indicating that such heterozygotes are likely to enjoy mimetic protection.

### Coordination of dominance

**Figure 2:**
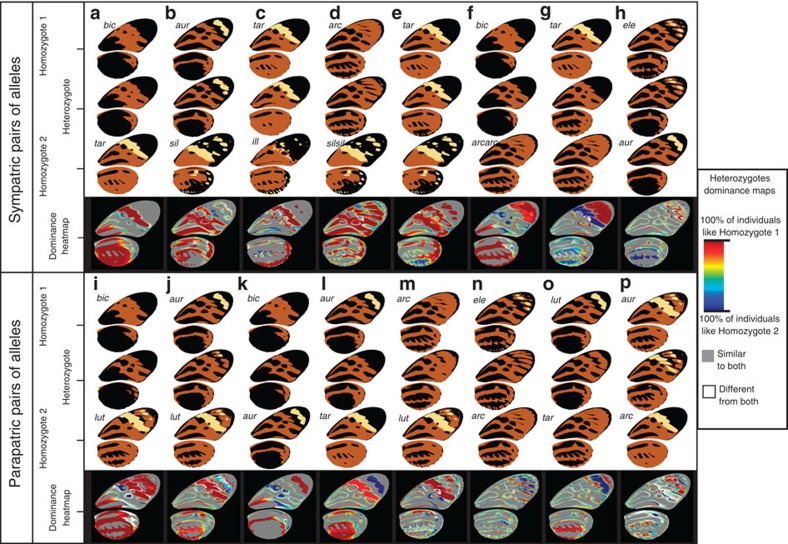
Dominance heat maps for all pairs of alleles. Upper and lower panels present sympatric (**a**–**h**) and parapatric (**i**–**p**) pairs of alleles, respectively. Each column (**a**–**p**) shows, for a given pair of alleles, the modal wing patterns of homozygous dominant (top), heterozygote (middle) and homozygous recessive (bottom) genotypes on a white background, and the corresponding dominance heat map on a black background. Each heat map pixel is coloured on a scale from red to blue representing the proportion of heterozygous individuals identical to one (red or blue), both (grey) or neither homozygote (white). Areas displaying a rainbow of colours are due to variations in patch size and shape among heterozygotes. Alleles are referenced with the first three letters of each mimetic form (*bic*: *bicoloratus*; *aur*: *aurora*; *arc*: *arcuella*; *ele*: *elegans*; *lut*: *lutea*; *tar*: *tarapotensis*; *sil*: *silvana*; *ill*: *illustris*).

Dominance coefficients measured in the previous section represent an estimation of dominance for the entire wing pattern. We then compared variations of dominance across the wing between sympatric and parapatric pairs of alleles using dominance heat maps (Fig. 2). Notably, we were interested in understanding whether the co-dominance of heterozygotes stemmed from the combination of patches with strong but opposite dominance directions (mosaic dominance), or from all elements of the wing showing similar but intermediate levels of dominance. Red or blue patches in the maps indicate pattern elements matching those of the parent homozygotes, and therefore represent the dominance or recessivity of each element. Grey indicates areas where heterozygotes are different from both of the parent homozygotes.

Six of the eight sympatric pairs of alleles produced heat maps mainly composed of red shades on a grey background (Fig. 2a–f) indicating the same dominance of one allele over the other for all pattern elements, in accordance with their high dominance coefficients (for example, *h*=0.95 for *bicoloratus/tarapotensis*, Fig. 2a). The remaining two maps from sympatric crosses showed both red and blue patches, indicating high dominance for individual elements but in different directions, resulting in intermediate values of dominance coefficient over the entire wing (for example, *tarapotensis/arcuella, h*=0.58, Fig. 2g).

In parapatric crosses, the heat maps (Fig. 2k–p) show that the variability of the overall dominance coefficient *h* is a result of a mosaic of independent dominance of the different pattern elements, producing intermediate overall values of *h* for six heterozygotes.

### Dominance of colours

**Figure 3:**
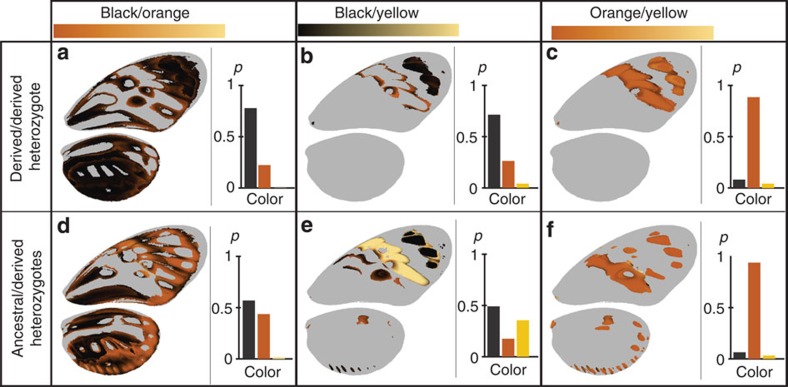
Colour hierarchy maps. These maps report, pixel-by-pixel, the proportion of heterozygous individuals expressing black, orange or yellow where their corresponding homozygotes express distinct colours. For visualization purposes, these proportions are translated into gradients of black, orange and yellow. Grey represents areas where no variation was observed for the corresponding pair of colour in homozygous genotype. For each pair of colour the probability of expressing each of the three colours over all pixels is summarized by a bar chart to the right of each map. Maps and bar charts calculated for: black/orange on the first column (**a**,**d**); black/yellow on the second column (**b**,**e**); orange/yellow on the third column (**c**,**f**). Colour hierarchy maps of derived/derived heterozygotes are represented on the first line, and the ones of ancestral/derived heterozygotes on the second line.

Then we analysed to which extent coordination and mosaic of dominance observed throughout the wings could be explained by a hierarchy in colour expression. By quantifying the wing colour patterns we were able to describe the pairwise dominance relationship between the three colours in heterozygotes (Fig. 3). Our results suggest that dominance relationships between the six alleles in the derived class (alleles carrying the inversion) is well explained by a transitive hierarchy of colour expression with black>orange>yellow. Across all relevant positions on the wing from crosses within the derived class, yellow was consistently recessive to orange; indeed, on the wings of heterozygotes, yellow was expressed in only 3.1% of the pixels for which homozygotes were yellow versus orange. Yellow was also consistently recessive to black (in heterozygotes, yellow was expressed in only 3.6% of relevant pixels). Orange was generally recessive to black (in heterozygotes, orange was expressed in only 29% of relevant pixels). Among alleles of the derived class, this transitive colour dominance hierarchy predicts the phenotype of heterozygotes in 79% of the wing areas where parental homozygotes display distinct colours (see Methods section). This indicates that both the coordination and the mosaic of dominance observed between alleles of the derived class were mostly a consequence of this colour hierarchy. Moreover, heterozygotes frequently express orange in 21% of pixels where homozygotes express black versus yellow (Fig. 3). Although our analysis considers this as a deviation from the colour hierarchy rule (under which black should be expressed), the expression of orange in black/yellow heterozygotes may reflect the intermediate position of orange in the hierarchy. The remaining part of the phenotypes unpredicted by this colour hierarchy was mainly caused by two different observations. First, intermediate patches size or shapes in heterozygotes caused deviations to the colour hierarchy predictions. Second, the variability of individual phenotypes compared with the modal wing patterns used for dominance quantification can also explain why in some wing areas (that is, white areas on the dominance heat maps), heterozygotes resemble neither homozygotes (on average 6.9% of the heterozygotes’ wing surface and especially along patch boundaries, for example, Fig. 2j).

By contrast, ancestral alleles (*illustris* or *silvana*, ancestral gene order) were fully recessive to derived alleles in all combinations tested, and independently of the colours displayed by the corresponding phenotypes. The dominance in such combinations is coordinated throughout the wing in favour of the derived allele, irrespective of the colours involved, and did not follow the hierarchy in colour expression inferred for alleles from the derived class. Across all derived/ancestral heterozygotes (that is, carrying an *illustris* or *silvana* allele), only 63% of colour expression followed the colour hierarchy. Permutation tests showed that the percentage of heterozygote phenotypes explained by colour hierarchy in derived/derived heterozygotes is significantly higher than in derived/ancestral heterozygotes (*P*=0.025; see Supplementary Methods). Moreover, derived/ancestral heterozygotes phenotypes were significantly better predicted by assuming the complete dominance of one allele, independently of colours than derived/derived heterozygotes phenotypes (nested ANOVA, *N*=472, F_1,471_=48.89; *P*<0.0001; see Supplementary Fig. 2). The difference mainly stems from the recessivity of the black colour of *silvana* and *illustris* to the yellow and orange colours controlled by alleles carried by the derived chromosome arrangement (see Supplementary Fig. 3). In heterozygotes between either *silvana* or *illustris* and a derived allele, the proportion of pixels where black was recessive to yellow was 37%, much higher than in heterozygotes for two derived alleles (4%). For black/orange relationships, the difference was twofold (43 and 22%, respectively). Overall, this supports the hypothesis that two distinct mechanisms of dominance determine the phenotype of heterozygotes, that is, (1) a hierarchy in colour expression within alleles from the derived class and (2) a complete dominance, independent of colour, associated with the 400-kb inversion.

## Discussion

Here we demonstrate that in *H. numata*, dominance relationships maximize mimetic resemblance in individuals heterozygous for sympatric alleles. In accordance with theoretical predictions^18^, this suggests that allele combinations that are frequent within natural *H. numata* populations are under selection against non-mimetic wing patterns in heterozygotes. This is in accordance with results in the mocker swallowtail *P. dardanus* in Africa, involved in Batesian mimicry with local models, where crosses within geographical races show stronger dominance relationships than inter-racial crosses^19^,^21^. Mimicry evolution in *Heliconius* and *Papilio* differs in many respects, as they belong to divergent butterfly families, mimic the patterns of distinct communities and have evolved supergenes in different genomic regions, carrying distinct genes controlling their mimetic patterns^9^,^25^,^35^. However, together with theoretical predictions^17^, our results support the hypothesis that in Müllerian mimicry too, selection maintaining mimetic polymorphisms can promote strong dominance between sympatric alleles, together with tight linkage that limits the production of intermediate, non-mimetic phenotypes. In apparent contradiction with the prediction of strong dominance in sympatry, two mimetic alleles, controlling the forms *tarapotensis* and *arcuella*, are co-dominant, despite being sympatric over a large part of their range. Remarkably, *tarapotensis/arcuella* heterozygotes enjoy the benefits of heterozygote-specific mimetic protection by joining a distinct mimicry ring. This exception therefore confirmed that the dominance between alleles at the supergene *P* is shaped by natural selection exerted by mimicry.

This selection on dominance might have influenced the diversification of mimetic alleles in the supergene *P* in *H. numata*. The genomic rearrangements observed at the supergene permitted to infer the relative age of the allelic classes and allowed us to understand the role of dominance in the evolution of the supergene *P*. The ancestral class of alleles includes the s*ilvana* and *illustris* alleles and another allele *laura*, which has the ancestral structural arrangement and a nucleotide variation close to the two other. All these three forms controlled by ancestral alleles coexist with other local mimetic forms of *H. numata* and are all strictly recessive to co-occurring, derived alleles^11^,^33^. This dominance of derived alleles to ancestral alleles is in accordance with ‘Haldane’s sieve’^5^,^6^,^7^, predicting that recent adaptive alleles are generally dominant to older ones. In other known supergenes, we noted that lower dominance also seems to be associated with older alleles, whose relative ages were inferred from structural differences such as inversions. Examples include the polygyny allele of the fire ant’s social supergene^12^; the Batesian mimicry allele of the *doublesex* supergene in *P. polytes*^9^ and a duplication of the *engrailed* gene in the *lamborni* allele of the supergene in *P. dardanus*^10^. This association between dominance properties and ancestral versus derived supergene alleles observed in different species suggests that dominance is an integral part of supergene architecture. Our results thus question the functional role of chromosomal rearrangements in promoting dominance of novel classes of alleles. The association of chromosomal rearrangements and dominance in *H. numata* could suggest that dominant alleles may have been captured by the inversions. However, dominance could also have evolved subsequently to the chromosomal inversions through linked dominance modifiers, in accordance with the observed association with the inversion. Geographically, *H. numata* supergene alleles belonging to the ancestral class generally replace each other in parapatry^33^, whereas differing derived alleles, with distinct chromosomal arrangements coexist with local ancestral alleles in all *H. numata* populations studied, across the species’ range^11^. Therefore, the dominance of derived over ancestral alleles may have contributed to the spread of the new morphs, and to their persistence as local polymorphisms in *H. numata.*

Our quantification of pattern variations using CPM has also allowed clarifying the mechanisms involved in the dominance between derived alleles. The phenotypes of heterozygotes carrying two derived alleles can largely be predicted by the operation, independently at every position on the wing, of a hierarchy between the colours controlled by the parental mimetic alleles. This hierarchy of colour expression in *H. numata* is different from other species in the genus, where yellow<white<black<red/orange prevails^30^. The development of colour patterns is generally assumed to respond to diffusion–reaction interactions among signalling molecules or morphogens^36^. Theoretical models indicate that mutations targeting diffusion capacities or sensitivity thresholds could drastically change the dominance relationships among alleles^28^. Changes in the properties of such signalling model could explain differences in the colour hierarchy observed in the *Heliconius* genus, including the dominance of black in *H. numata*. This evolution of colour hierarchy in *Heliconius* genus seems adaptive, since it allows the widespread co-occurrence of derived alleles displaying different levels of melanism, for which heterozygotes fit local mimicry rings.

**T**his hierarchy of colour expression does not predict the strong dominance of derived alleles over the ancestral ones. Indeed, the phenotypes of derived/ancestral heterozygotes rather fit the prediction of a dominant allele with coordinated effects across all functional elements controlled by the supergene, independently of colours. This novel dominance mechanism could have arisen from several possible molecular processes. This complete dominance of the derived alleles over ancestral ones could have evolved by changes in *cis*-acting elements of the functional genes within the inversion, for instance, increasing the overall expression of all functional transcripts, as observed at the *doublesex* supergene in *P. polytes*^9^. Alternatively, *trans*-acting elements could be associated with the inversions, downregulating ancestral alleles. *Trans*-acting control of dominance through small interfering RNA has been described for the polymorphic self-incompatibility locus of *Brassica*^37^, and provides a molecular mechanism by which dominance can evolve independently of the phenotype associated with each allele.

**Figure 4:**
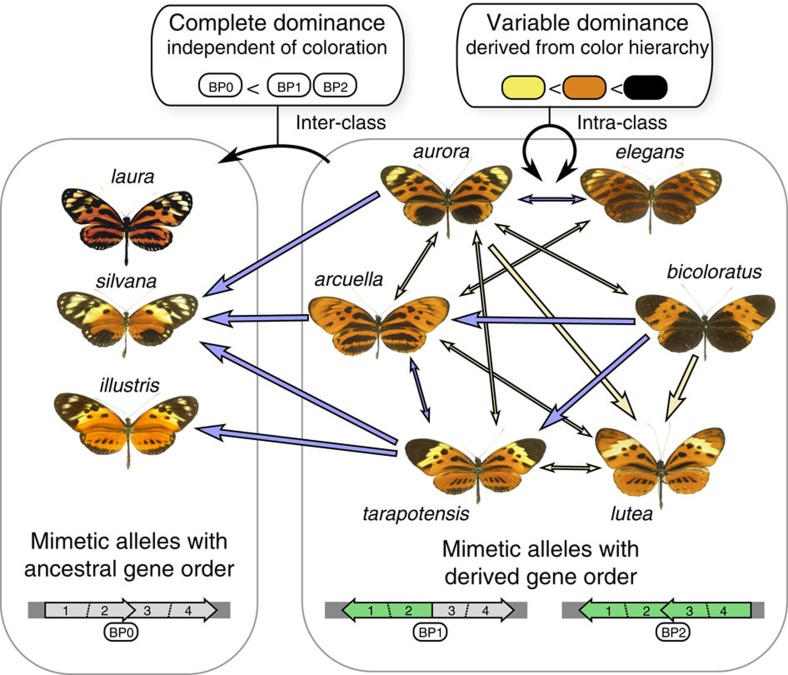
Relationships between dominance mechanisms and inversion polymorphism at the supergene. Arrows between butterflies represent all dominance relationships measured between and within the two allelic classes (blue: sympatric pairs; yellow: parapatric pairs). Thick single-headed arrows indicate strong dominance (*h*>0.8), thin double-headed arrows indicate co-dominance (0.5<*h*<0.8). Structural variation at the supergene is indicated at the bottom (left: ancestral gene order; right: novel, derived gene orders BP1 and BP2 sharing the first, 400 kb inversion.

Altogether, our results provide two lines of evidence indicating that dominance might have evolved in *H. numata*. First, we described a colour hierarchy different in this species compared with other species in the genus. Second, we showed that dominance properties in *H. numata* are determined by the combination of two distinct mechanisms: on one hand, mosaic dominance between alleles of the derived classes, emerging from a hierarchical expression of colours, and on the other hand, a complete dominance of the derived class of alleles over the ancestral class (Fig. 4). These two mechanisms and their association with the sequence of evolution of inversions at the supergene suggest that a mechanism of complete dominance has also evolved during the diversification of *H. numata* mimicry alleles, promoted by balancing selection. Our results stress the importance of selection acting on dominance in any locus under strong balancing selection. We also highlight the need to investigate the joint evolution of dominance and structural variations associated with these polymorphic loci.

## Methods

### Samples

Controlled crosses were performed between butterflies collected in different natural populations located close to the city of Tarapoto (San Martin district) in Eastern Peru (Supplementary Fig. 4 and Supplementary Table 1). In total, 843 individuals were analysed. Allelic combinations were classified as sympatric or parapatric based on the distribution of wing-colour-pattern phenotypes observed in natural populations^32^ (see Supplementary Methods for more details).

### Genotyping

Genomic DNA was extracted from frozen thorax tissue using the Qiagen DNeasy blood and tissue extraction kit. The alleles of the supergene *P* were genotyped using three microsatellite loci (*P3*, *P10* and *P11*) located within the supergene *P* genomic region (see Supplementary Methods and Supplementary Table 2 for more details). Genotypes are available as Supplementary Data 1.

### Phenotype characterization by Colour Pattern Modelling (CPM)

The novelty of the CPM method rests on three aspects (Supplementary Fig. 5). First, CPM uses the entire wing surface for pattern quantification without breaking down the wing pattern into elements. This avoids making assumptions on patch homology across individuals or restricting the analysis to specific types of variation. Second, CPM models explicit the distribution of colours across the wing, by treating colours as classes independent of minor colorimetric variations. This model aims to describe efficiently the variation in patch boundaries. Finally, quantifying fine features of colour patterns requires setting a proper homology between wing positions (that is, pixels) across individuals. Wing images were therefore aligned based on the similarity of their pattern to a model wing. This alignment maximizes pattern-matching across individuals, and, therefore, focuses the pixel-by-pixel analysis on variation in the relative shape and position of pattern boundaries.

Briefly, CPM was implemented as follows. For each specimen, dorsal and ventral sides of forewings and hindwings were photographed using standardized conditions (Supplementary Methods). Wing outlines were automatically and precisely extracted from the background^38^,^39^. To partition the wings into the different colour types, a colour categorization was first performed without constraining the number of colours (Supplementary Methods)^40^,^41^,^42^. Images were simplified by size reduction and region merging^43^. Colours were imputed automatically using a threshold on RGB values, followed by manual checking to correct for errors, which were mainly due to minor damage to wings. Starting from images with continuous RGB values, pattern was extracted by classifying colour, which enables tracking the distribution of similar colour types within and between wings (see also ref. 44). Extracted colours were set to correspond to one of the three major colours in *H. numata* wing patterns (that is, black, orange or yellow). Wing images were then aligned based on the pattern itself, independently of the wing structural features such as venation and outline. Forewings and hindwings were aligned separately. A wing model averaging all wing images was built after an initial alignment based on wing outlines. Each wing image was then transformed by adjusting translation, rotation and scale in order to maximize similarity^45^,^46^,^47^ to this wing model, which was recursively redefined at each iteration. The alignment implementation was based on the Insight Segmentation and Registration Toolkit^48^,^49^,^50^,^51^,^52^,^53^. CPM recursively maximizes the match of the whole-wing pattern over all individuals, and therefore defines pattern-based positional homology across the wing surface allowing a pixel-by-pixel analysis of pattern variation.

Wing-pattern phenotypic variation was summarized by principal component analysis (PCA)^54^ (Supplementary Fig. 6), with each non-background pixel common to all stacked wing images being considered a trait^55^. Only the PCA components explaining more than 1% of the variance (that is, 15 first components) were retained to perform our analyses in the PCA space. Multivariate analysis of variance on these components showed that genotypes at the wing-patterning supergene were significantly discriminated (*N*=648, F_15,625_=149,860, *P*<0.0001), confirming the validity of our approach. This was confirmed by the leave-one-out cross-validation fraction of the linear discriminant classifier, which showed that three components were sufficient to correctly classify 100% of homozygotes for each allele. Phenotypic distance between pairs of homozygotes was considered to be the distance between the average positions of each group of homozygous individuals.

### Quantification of dominance

For a given quantitative trait *T*, the dominance (*h*) of allele a relative to allele b was computed using this equation: 
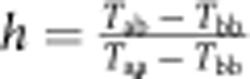
 where *T*_ab_, *T*_aa_ and *T*_bb_ represent the average trait values for heterozygotes ab, and homozygotes aa and bb, respectively. Strict dominance of a with respect to b corresponds to *h*=1 and intermediate values (*h*~0.5) represent co-dominance. The trait *T* used here to quantify colour pattern variation was derived from the relative proportion of wing area shared between the heterozygote and either homozygote. For a given homozygous genotype, a modal wing pattern was built, calculated by setting each pixel to its modal colour value, that is, shared by the most specimens with this genotype. For each pair of alleles, the trait *T* was then calculated as the number of pixels in the heterozygotes that were similar to one homozygote modal pattern and different from the other, normalized by the wing surface (in pixels). This calculation could be performed on 93.1% of the wing on average, because a small proportion of the wings of heterozygotes matched neither homozygote modal wing colours. All dominance coefficients measured were normalized so that allele a (equation above) corresponded to the more dominant allele, constraining *h* values to range between 0.5 and 1. An alternative estimate of the colour pattern trait was computed based on a linear discriminant analysis, and it gave values that were highly correlated with the surface-based estimate used here (Supplementary Figs 7 and 8).

### Dominance and colour hierarchy

Dominance heat maps were generated for each heterozygous genotype to visualize how dominance varies across the wing. For each pair of supergene alleles, heat maps represent, on a pixel-by-pixel basis, the proportion of heterozygous individuals for which the colour was identical to one, both or neither homozygote.

To visualize the consistency of the model of colour hierarchy across the wing, we constructed wing maps reporting the colour expressed by heterozygotes when the corresponding homozygotes show distinct colours. Maps were not constructed for a pair of alleles, but for a pair of colours. For each pair of colours, at a given pixel position, we considered all pairs of supergene alleles showing the colour difference between the modal wing patterns of homozygotes, and computed the proportion of heterozygous individuals expressing each colour. The map obtained, termed a ‘colour hierarchy map’, translates these proportions into gradients of black, orange and yellow, visually showing the consistency of the hierarchy across the wings.

To estimate the dominance of one colour to another colour, we computed the probability of expressing the dominant or the recessive colour in heterozygotes throughout all selected pixels for all genotypes. Here the expression of one colour was defined as its probability of being expressed in heterozygotes. Finally, we estimated the proportion of the phenotype of heterozygotes explained by colour hierarchy by computing the overall proportion of pixels conforming to the general hierarchy across the three colours.

## Author contributions

Y.L.P., V.L. and M.J. designed the experiment, M.J. performed the controlled crosses, V.L. took the standardized pictures of butterfly wings, A.W., M.C. and F.P. designed and performed the genotyping, Y.L.P. built the CPM method and performed all analyses. V.L. and M.J. supervised the analyses. Y.L.P., A.W., V.L. and M.J. wrote the manuscript.

## Additional information

**How to cite this article:** Le Poul, Y. *et al*. Evolution of dominance mechanisms at a butterfly mimicry supergene. *Nat. Commun.* 5:5644 doi: 10.1038/ncomms6644 (2014).

## Supplementary Material

Supplementary Figures, Tables, Methods and References Supplementary Figures 1-8, Supplementary Tables 1-2, Supplementary Methods and Supplementary References.

Supplementary Dataset 1 Genotype table
