# Supplementary material for: Evolution of dominance mechanisms at a butterfly mimicry supergene
Source: Nat Commun. 2014 Nov 27;5:5644. doi: 10.1038/ncomms6644 (PMC4263167; doi:10.1038/ncomms6644)
Supplement: Supplementary Figures, Tables, Methods and References — Supplementary Figures 1-8, Supplementary Tables 1-2, Supplementary Methods and Supplementary References. [file ncomms6644-s1.pdf]

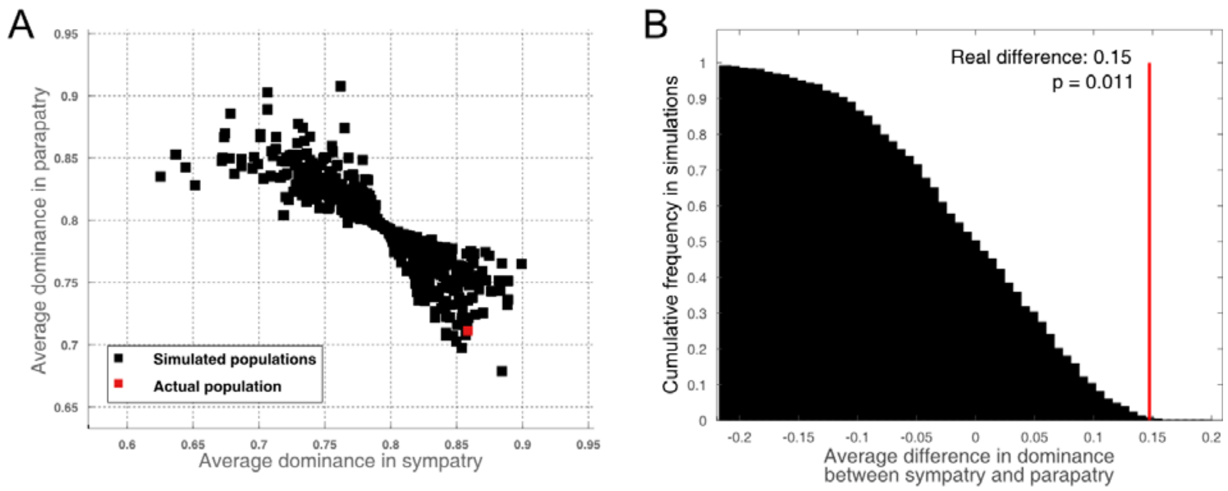

Supplementary Figure 1. Simulated distribution of average dominance in sympatry and parapatry. A. Scatter plot of the simulation results. B. Decreasing cumulative frequency of the difference. The *p* value corresponds to the proportion of cases in the 10,000 permutations for which differences observed between average dominance in sympatry and parapatry is higher than actually measured. A and B show that the difference between average dominance in sympatry and parapatry lies on the extreme of the simulated distribution.

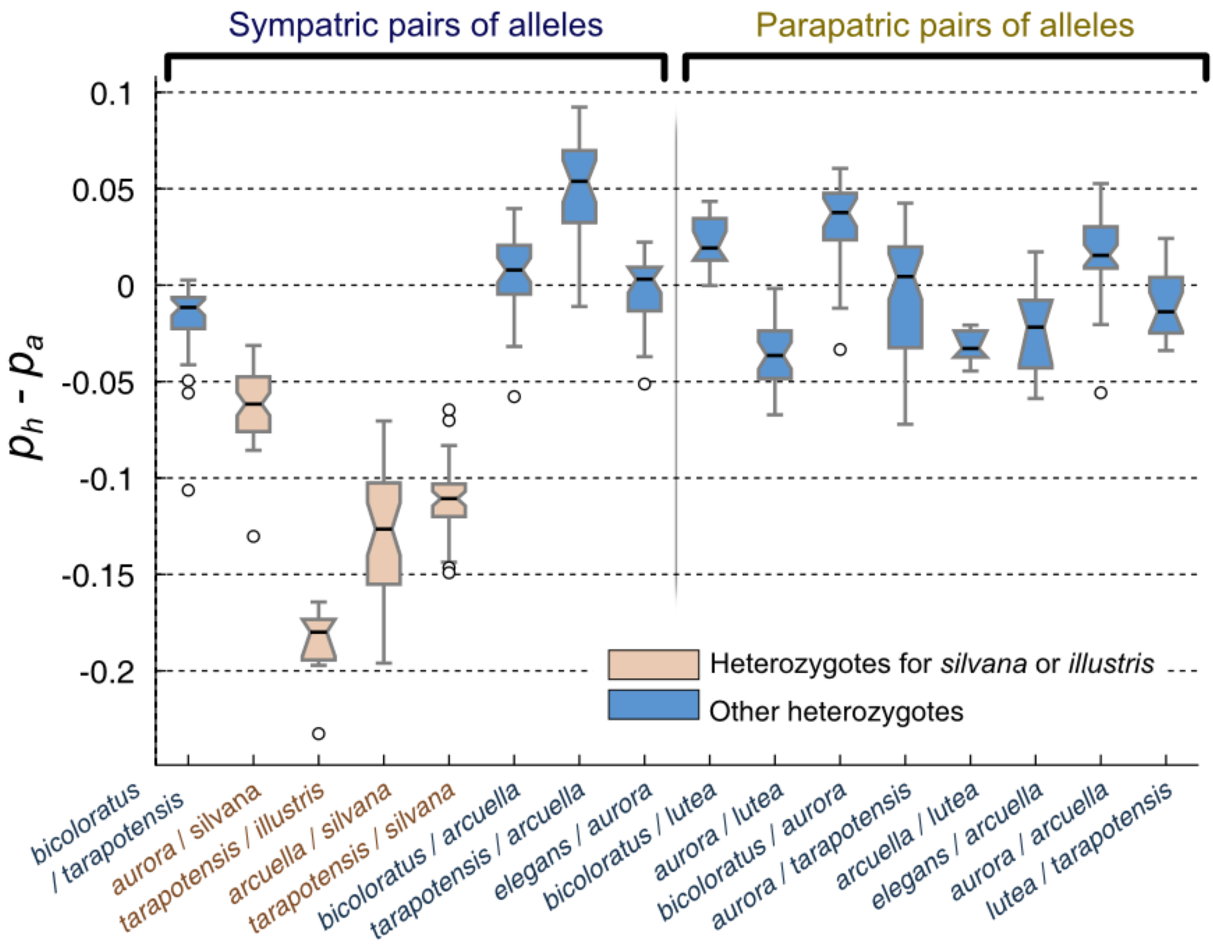

Supplementary Figure 2. Difference between the proportion of the wing similar to a model wing generated by colour hierarchy and to the dominant allele. Box plot with median, first, third interquartile range, extreme value, and outliers represent this difference for each heterozygous genotype (N = 472). Heterozygotes carrying a *silvana* or *illustris* allele display a higher proportion of wing area similar to the dominant homozygote than to the hierarchy model. For others heterozygotes, the difference are close to zero indicating that the hierarchy model predicts the direction of dominance for all of them.

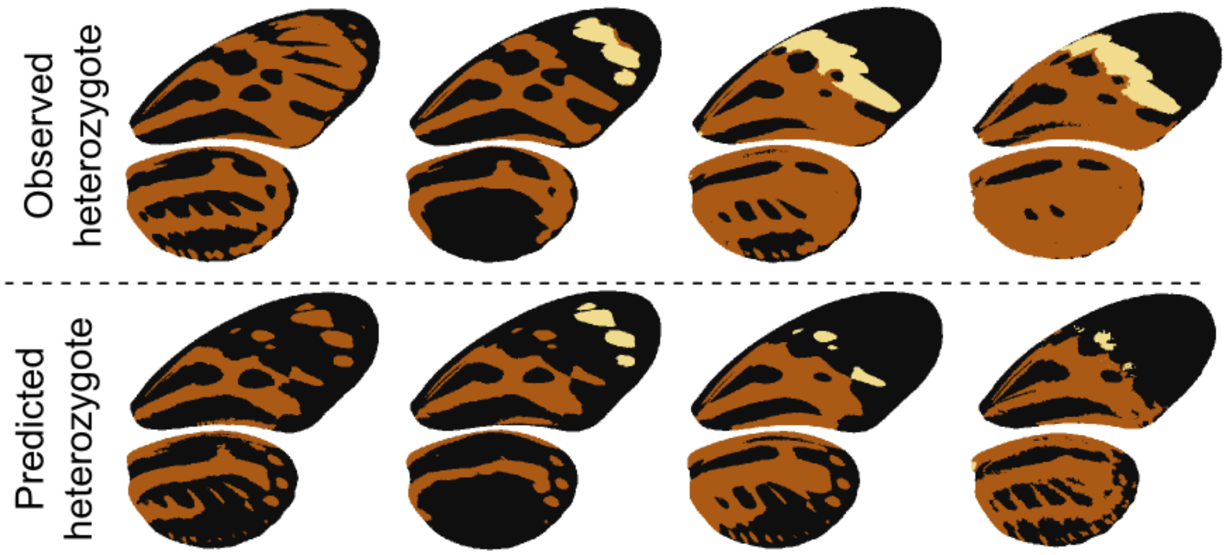

Supplementary Figure 3. Predicted and observed phenotypes of heterozygotes for the *silvana and illustris* alleles. Predicted phenotypes are constructed by applying the colour hierarchy rules for *arcuella*, *aurora*, and *tarapotensis* in the first, second, and third columns, respectively.

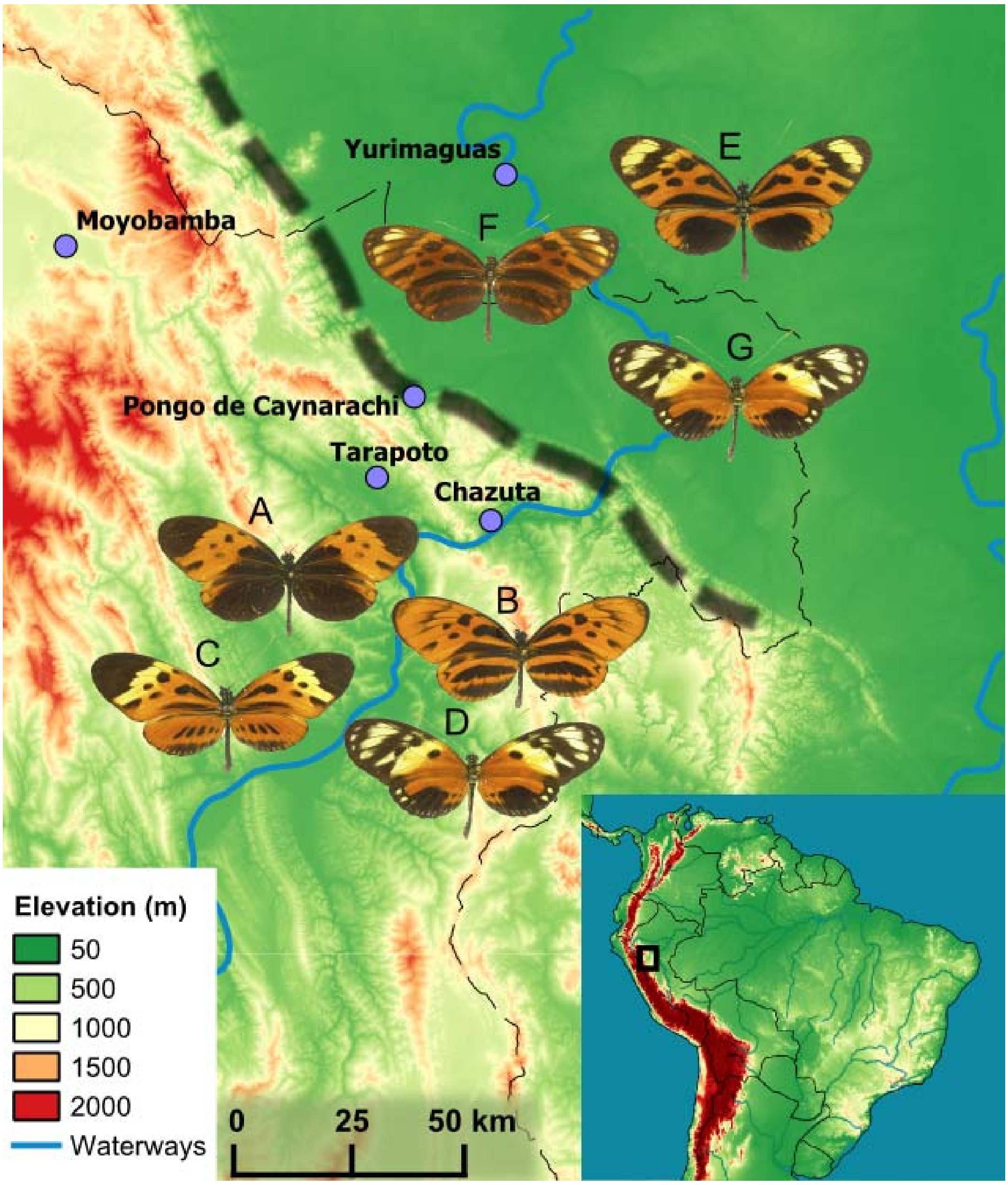

Supplementary Figure 4. Spatial distribution and sympatry of the mimicry alleles in the Andean foothills of Eastern Peru. This region surrounding the city of Tarapoto, San Martin, is divided into two adjacent biogeographic areas differing in the set of coexisting mimetic forms, determined by specific supergene alleles: Andean valley (west: forms A, B, C, D and E) and Amazonian lowlands (east: forms E, F and G). Pairs of alleles are defined as sympatric when they are naturally frequent and coexist in the same region. A: *bicoloratus*; B: arcuella; C: *tarapotensis*; D: *lutea*; E: *silvana*; F: *aurora*; G: *elegans*.

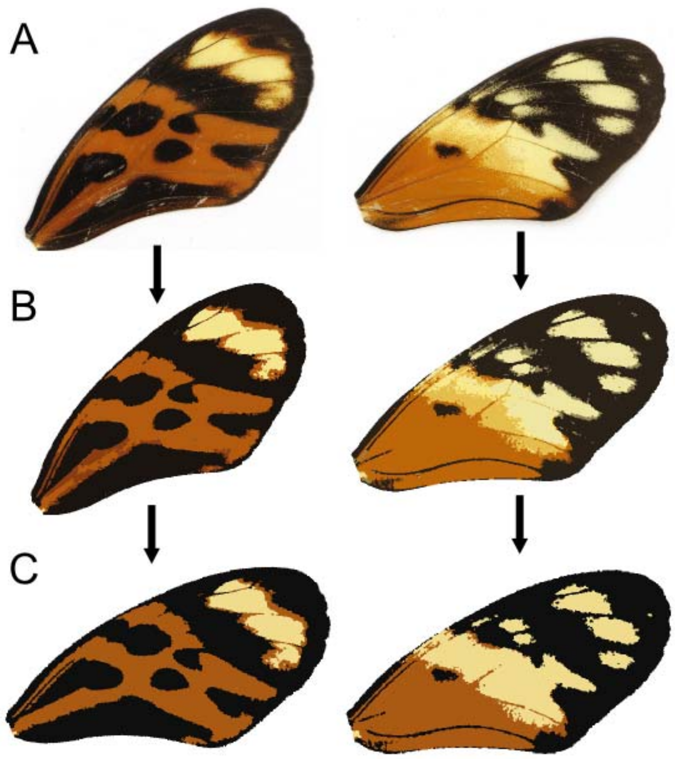

Supplementary Figure 5. Colour pattern analysis. A: Wing extracted from natural image; B: automatic colour attribution; C: attribution of each pixel to one of the 3 scale types and alignment of the wings.

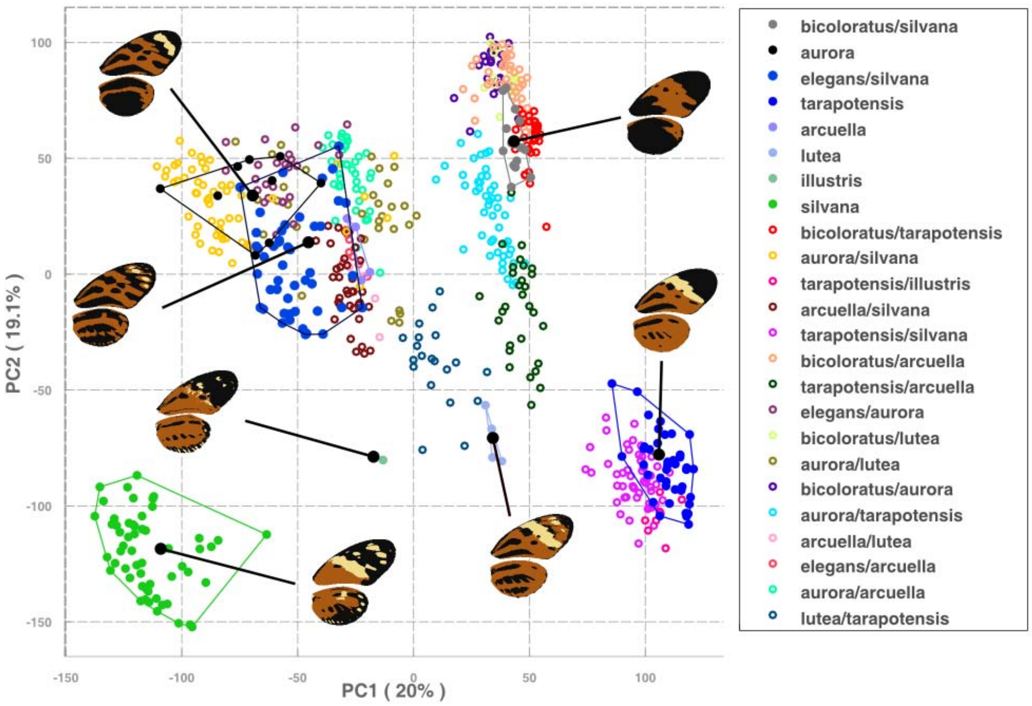

Supplementary Figure 6. Morphospace covered by the 648 individuals, representing 23 genotypic combinations of 8 alleles at the supergene *P*. This graph was obtained by Principal Component Analysis using each position of the superimposed wings as a trait. Filled circles represent homozygous individuals, whereas open circles represent heterozygotes. Axes 1 and 2 of this PCA explain 34.2% of the overall variance.

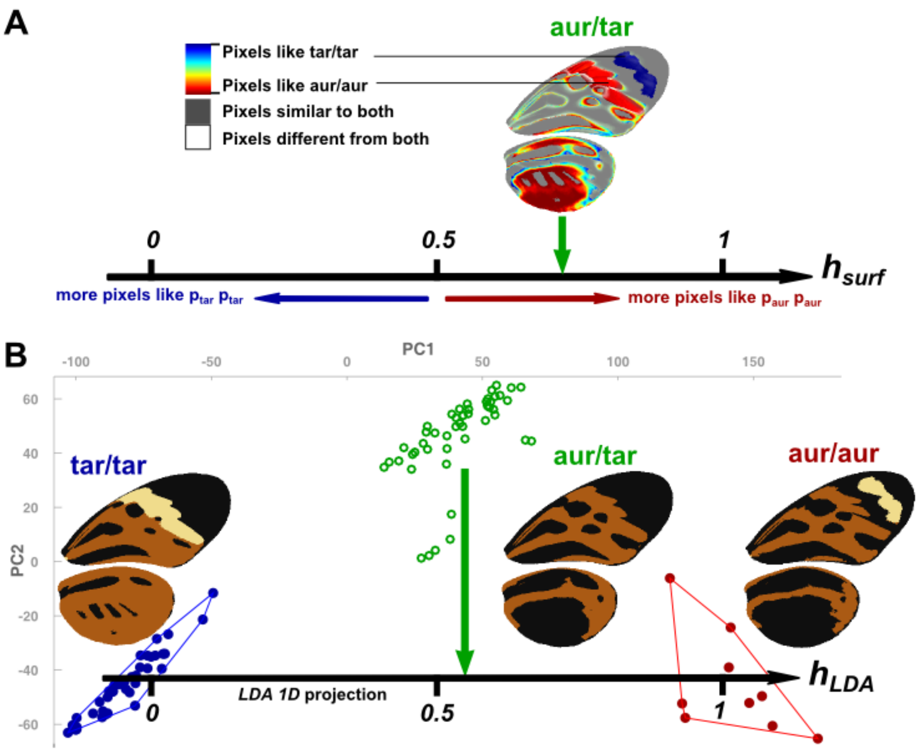

Supplementary Figure 7. Comparison of the two types of traits used to estimate the dominance coefficient. (A). A two-class discriminant vector is calculated from the two homozygotes. The projection of the heterozygous phenotype on this vector is used to calculate the dominance coefficient h_{lda}. (B) The proportion of pixels resembling one of the homozygotes, normalized by the wing surface is used to calculate the dominance coefficient h_s.

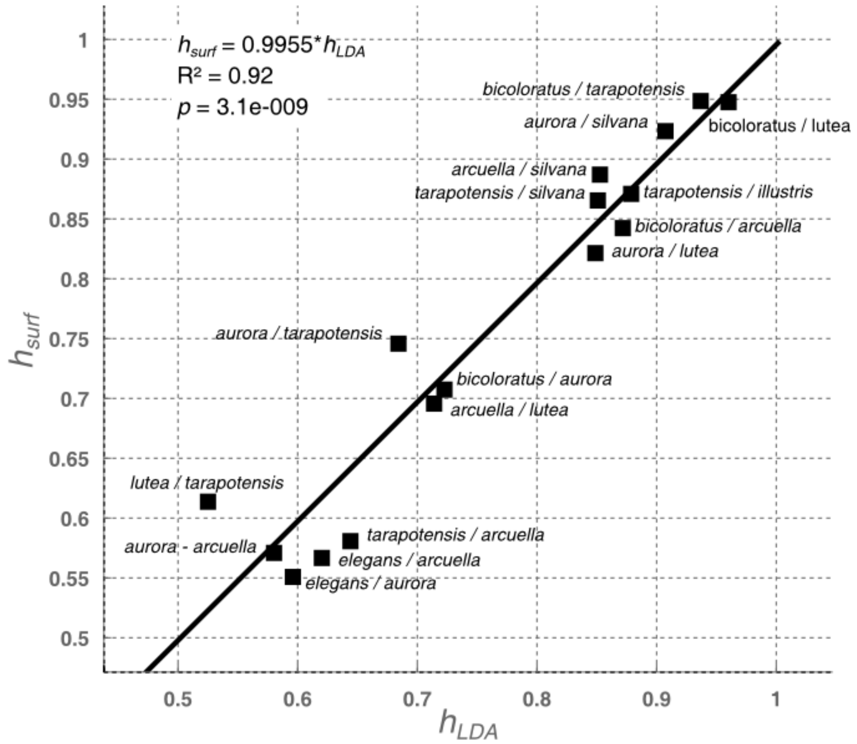

Supplementary Figure 8. Comparison of the two estimates of the dominance coefficient h, based on proportion of surface (h_{surf}) and on Linear Discriminant Analysis (h_{LDA}). Scatter plot show the correlation between average dominance for both estimates.

Supplementary Table 1. Table of crosses.

Class	Genotypes	Number of	Number of	Geographic
Heterozygotes	*bicoloratus/tarapotensis*	50	5	Sympatric
	aurora/silvana	63	4	Sympatric
	tarapotensis/illustris	11	1	Sympatric
	arcuella/silvana	52	9	Sympatric
	tarapotensis/silvana	76	7	Sympatric
	bicoloratus/arcuella	49	4	Sympatric
	tarapotensis/arcuella	35	2	Sympatric
	elegans/aurora	28	2	Sympatric
	bicoloratus/lutea	17	1	Parapatric
	aurora/lutea	32	2	Parapatric
	bicoloratus/aurora	31	4	Parapatric
	aurora/tarapotensis	59	5	Parapatric
	arcuella/lutea	13	1	Parapatric
	elegans/arcuella	16	3	Parapatric
	aurora/arcuella	48	4	Parapatric
Homozygotes	*bicoloratus/silvana* (*)	22	4	-
	aurora/aurora	12	2	-
	elegans/silvana (*)	63	6	-
	tarapotensis/tarapotensis	61	9	-
	arcuella/arcuella	10	3	-
	lutea/lutea	7	1	-
	illustris/illustris	1	1	-
	silvana/silvana	67	8	-

Supplementary Table 2. Sequences of the primers used to amplify the three microsatellite loci located in the supergene *P* region. Note that the forward primer contains the reverse complement of the *M13* tail which appears in bold font.

Microsatellite name	Primer name	Primer sequence (5' to 3')
**P3**	P3F	**CACGACGTTGTAAAACGAC**CAGCACTTTATCTAGAAATTATATAGA
	P3R	GCAHTGCAAAGGATGGTAATG
**P10**	P10F	**CACGACGTTGTAAAACGAC**TCGTAGGTATTCGGAGAACG
	P10R	CTCTGCGTTCCCATTAAGAA
**P11**	P11F	**CACGACGTTGTAAAACGAC**ACCACATGGGGGTCTAAAGT
	P11R	CGAACTTCCGTTGCACTCT

SUPPLEMENTARY METHODS

1. Controlled crosses. 33 independent broods were obtained from controlled crosses between wild-caught individuals. In total, 843 individuals were analysed (600 heterozygotes and *243* homozygotes), with the heterozygous genotypes in 8 sympatric combinations and 8 parapatric combinations (table S1 and Supplementary Figure 4). For each specimen, the body was separated from the wings, snap-frozen and subsequently used for DNA extraction. Wings were stored in individual glassine envelopes and subsequently used for wing pattern digitization and analysis. Individuals for which only one wing could be analysed (*e.g.* due to wing damage) were therefore excluded from calculations performed on both wings.

Our crosses do not include *elegans* or *bicoloratus* homozygotes, due to their rareness in wild populations and the difficulties in breeding these genotypes. In these two cases, we used heterozygotes with the *silvana* allele as a substitute. This substitution had little effect on the results because *silvana* is the most recessive allele in the dataset. Indeed, *silvana* heterozygotes are very close or even undistinguishable from the corresponding homozygotes in the PCA phenotypic space (Supplementary Figure 6). This is the case, for instance, for the *aurora* allele (the closest to *elegans* in terms of phenotype). Furthermore, heterozygotes with the *bicoloratus* allele all fall very close to each other in the PCA space indicating its strong dominance over other alleles. Among all heterozygotes for the *bicoloratus* allele, *bicoloratus*/*silvana* was the furthest from all other homozygotes, therefore appearing as the most conservative substitute for the *bicoloratus* homozygous genotype. Finally, as expected for the top dominant allele, *bicoloratus* homozygotes are rare in natural populations where nearly all specimens with the *bicoloratus* phenotype are heterozygous[1]. Selection is therefore likely to act on the *bicoloratus* allele mainly in heterozygous individuals.

2. Genotyping. Genotyping was carried out using three microsatellite loci (*P3, P10* and *P11*) amplified using primers described in Table S2. The association between microsatellite length polymorphism and wing pattern alleles of the supergene was checked using broods where wing-pattern genotypes were already known or were unambiguous[2]. Polymerase Chain Reaction (PCR) was carried out in a 10 µl mix composed of 2µl gDNA,1x DreamTaq buffer, 200µM dNTPs, 0.5µM universal M13 primers labelled with YAKYE, AT565, AT550 or FAM from Eurofins, 0.5 µM reverse primer, 0.01 µM forward primer and 0.25units of DreamTaq. PCR performed using a Techne TC-5000 thermocycler with 2 min at 94°C, followed by 35 cycles with 30 sec at 94°C, 30

sec at 53°C and 30 sec at 72°C, and a final elongation phase at 72°C for 12 minutes. Fragment analysis was run on an Applied Biosystem 3130 genetic analyser, using SoftGenetics GeneMarker Version 2.4.0 for analysis.

3. Picture analysis by Colour Pattern Modelling (CPM). In order to quantify wing colour pattern variation, we built appropriate procedure able to analyse patterns comprehensively from pictures of wing, referred to as Colour Pattern Modelling (CPM), and summarized in Supplementary Figure 5.

Normalized photographs. First, for each specimen, both sides of each forewing and hindwing were photographed in normalized light conditions (CIE Standard Illuminant D50), with a high colour rendering light source (Philips Master TL-D 90 Graphica pro). A scale indicator was included in each picture, and the white balance was normalized. A Nikon D90 digital camera with a Nikon micro 105/2.8G ED VR lens was used to capture high resolution images with accurate colour rendering. The right wing was analysed, unless extensive wing damage precluded this, in which case the left wing was mirror-transformed during the analysis. In *H. numata*, ventral and dorsal sides display a similar colour pattern, with the exception of the regions where hindwings and forewings overlap, which are composed of modified grey scales on their ventral or dorsal side, respectively, thereby replacing colour pattern expression. Therefore, we used the dorsal side of forewings and the ventral side of hindwings to perform the analysis on the whole pattern for both forewing and hindwing.

Wing extraction. The first step of CPM relies in identifying and extracting outline of wings on pictures. Wings were automatically detected in the images using their colour difference with the homogenous white background, and were then precisely extracted using the marker-based watershed transformation [3] along the image colour gradient [4]. This segmentation method finds the maximum intensity of the colour transition between the marked wings and the marked background, which was then considered to be the wing outline.

Colour number reduction and colour attribution. Then, the pattern was modelled by considering explicitly the mosaic distribution of colour across the wings, which allows describing efficiently the variation in patches boundaries. A set of discrete colours characteristic to each wing was first identified using an algorithm based on colour histograms [5,6]. After simplifying the spatial structures of wing images [7], pixels were then attributed to each colour (black orange and yellow) using a simple threshold.

Colours classes were extracted from the histogram by the following procedure. First; to smooth the colour distribution on the histogram and simplify colour histogram processing, the image was projected from the 3-dimensional RGB colour space to a 2-dimensional (2D) colour space, where dimension 1 corresponds to luminance (the Y component in the Ycrbr colour space), and dimension 2 to the major colour variation axis using all wing images. This projection preserved about 97% of colour variance in the images. A 2D-histogram, representing the distribution of pixel colour, was computed in the same 2D-projected colour space for all wings. Each separate colour on the wing was defined as a local maximum on the 2D-histogram. These local maxima are always numerous because of the complexity of the natural image. To prevent over-segmentation, minor peaks were automatically removed by consideration of their proximity to and separation from neighbouring peaks. We performed a watershed transformation on the additive inverse of the 2D-histogram to partition the colour space among the major peaks [8]. At the end of this process, each wing could be associated with a set of characteristic colour partitioning the colour space and accounting for the colour variation actually present on the wing RGB image.

In order to preserve the patch structure of colour patterns, we also performed a routine to merge neighbouring pixels of homogenously-coloured regions in the images [7]. The scale indicator within each image was first used to rescale images to an output length of around 512 pixels, leading to a mean spatial resolution of 10.9px/mm. Each reduced image was then transformed into a mosaic of homogeneously-coloured spatial zones. A watershed transformation of the image colour gradient was used to carry out the mosaicking [4]. Each homogenously-coloured region was then attributed a colour according to the classification given by the segmented histogram.

Finally, the attribution to the different colours (black, orange, and yellow) was done automatically using a threshold on RGB values, followed by manual checking to correct errors, which were mostly due to minor damage to parts of the wings, resulting in the final segmented image (Supplementary Figure 5).

Alignment. For the wing images to be efficiently comparable pixel by pixel, a proper homology of pixel positions was needed. Since the pattern is composed by multiple elements varying across the different morphs, the differences in patterns mostly relies in differences in position and shape of these elements relative to the others. These differences can be described efficiently only if patterns are aligned in a way that they display a maximal match relative to each other. This match was obtain by transforming each set of processed images into a common coordinate system

which maximize similarity between each wing pattern to a wing pattern model (*i.e.* the ‘mean pattern' of all individuals), treating forewings and hindwings separately. Similarity was measured by the Mattes implementation of mutual information metric [9–11], which is minimal when colour patches are aligned in an optimal compromise. The one+one evolutionary optimizer [12,13], implemented within the ITK free image proceeding library in C++ [14–17], was used to find the scale, rotation and translation parameter set that minimized this mutual information value. This procedure created an initial registration set based on wing shape, which allowed generation of the wing pattern model. Each wing was then recursively aligned to the model, until the variance of the metric stabilized (variance varying less 1% [18]). At the completion of this process, all wings could be considered to be positioned in the same physical space, with pixel locations and colour values among wings being comparable among all individuals.

Validation of CPM method. The phenotypic diversity of wing patterns in all broods studied was explored by Principal Component Analysis (PCA), as a classical dimensionality reduction approach for stacks of images. Each non-background pixel common to all stacked wing images was considered as a trait in the PCA [19]. MANOVA on those selected components showed that genotypes were significantly discriminated, confirmed by the leave-one-out cross-validation fraction of the linear discriminant classifier. As few as 3 components were sufficient to classify at 100% rate the homozygotes for each phenotype. This high rate of classification confirms that the CPM method characterizes the complex colour patterns well. The two first components of the PCA performed on the wing pattern of 648 individuals are presented in Supplementary Figure 6.

Dominance estimation. To quantify dominance, we used the so-called dominance coefficient h. For a given quantitative trait T, the dominance of allele a relative to allele b is given by the equation: $h = \frac{T_{ab} - T_{bb}}{T_{aa} - T_{bb}}$ where Tab , Taa and Tbb represent the mean trait values for heterozygotes ab, and both homozygotes aa and bb, respectively. Strict dominance of *a* with respect to *b* corresponds to h = 1, strict recessivity is represented by $h = 0$, and intermediate values ($h \sim 0.5$) represent co-dominance. All dominance coefficients were normalized so that allele *a* corresponds to the more dominant allele. This measure of dominance is based on a one-dimensional value of *a* trait. Because of the high dimensionality of wing characters, *a* dominance coefficient was estimated using two alternative metrics of T leading to two measures, h_{surf} and h_{LDA} (Supplementary Figure 7).

For the first estimation of dominance, h_{surf}, presented in Supplementary Figure 7B, the trait T used here to quantify the colour pattern variation was derived from the relative proportion of wing area shared between the heterozygote and either homozygote. For a given pair of alleles, a mean wing pattern was built for each homozygote, calculated by setting each pixel to the colour shared by the highest number of specimens bearing this genotype. For each trio of genotypes considered, we calculated the number of pixels of the heterozygote that were similar to one homozygote and different from the other, normalizing by the wing surface (in pixels). This calculation could be performed on 93.1% of the wing area on average, because a small proportion of the heterozygote wing area matches neither homozygote models.

The second estimation of the dominance coefficient, h_{LDA}, presented in Supplementary Figure 7A, was based on the projection of the phenotypes on a two-class linear discriminant vector which best separates the two corresponding homozygous phenotypes described by the 15 first components of the PCA space.

4. Validation of the dominance estimation. In the main document, only the h_{surf} was presented for simplicity, but the second measure of the coefficient of dominance, h_{LDA}, validated the quantification approach. These two measures (h_{surf} and h_{LDA}) were indeed highly correlated (Pearson Correlation, N = 427, $R^2 = 0.92$, $p < 0.0001$), indicating their consistency (Supplementary Figure 8.).

5. Sex and brood effect. To check whether the sex or the genetic background of the individuals tested could bias the differences in dominance in sympatric and parapatric crosses, we tested the effect of these two factors on the dominance coefficient *h*.

First, females show slightly but significantly higher dominance values than males (Ttest, N = 472, $t = -2.51$, $p = 0.025$; for h_{surf}; $t = -3.26$, $p = 0.0058$ for h_{lda}), with a mean difference of *h* between males and females for each genotype of 0.027 and 0.022, respectively. The difference between male and female dominance coefficients is very low compared to the differences in dominance coefficients between sympatric and parapatric crosses, and thus has a negligible influence on our results.

Second, testing the effect of brood on the dominance coefficient reveals that the genetic background had no effect on dominance measure (Nested ANOVA, N = 472, $F_{27,471} = 0.44$; $p = 0.97$).

6. Significance of sympatry effect. To confirm that dominance was higher in sympatric crosses, we compute the significance of the difference between the average dominance of sympatric crosses *vs* parapatric crosses by a permutation test on sympatric relationships. Because these permutations aimed at testing whether dominance was higher in sympatry than in parapatry, the *tarapotensis/arcuella* genotype was excluded since this genotype shows heterozygote-specific mimicry and therefore does not match the general prediction that dominance should be higher in sympatry due to positive selection for dominance of one allele.

These permutations were performed by randomizing the location of alleles in one of the two populations present in our study and then inferring sympatric relationships. These simulations were carried out while keeping allele number per population identical to the real dataset. The differences observed between average dominance in sympatry and parapatry lies on the extreme of the possible values in the 10,000 permutations. These permutations confirmed a significant effect of sympatry on dominance ($P = 0.011$), highlighting the positive selection acting on dominance (see Supplementary Figure 1).

7. Mechanisms of dominance. We used permutation tests to evaluate the significance of the difference between the percentage of colour expression explained by colour hierarchy for heterozygotes with one of the alleles from the ancestral class, and for heterozygotes between alleles of the derived class. We generated permutations by randomizing the within-class vs between-class attribute of heterozygotes. These permutations showed the significance of the observed difference ($p = 0.025$).

To test for an association of allelic classes with alternative models of dominance (*allelic dominance* vs. *colour hierarchy*) we compared the match of heterozygotes to the phenotypes predicted by either model. For each heterozygote, the phenotype predicted by the *allelic dominance model* was represented by the phenotype of homozygotes for the more dominant allele, whereas the phenotype predicted by the *colour hierarchy model* was generated by applying the hierarchy rules to the corresponding pair of homozygotes. We then computed the proportion of the wing of actual heterozygous wing matching the *colour hierarchy model* (p_h) and the proportion of the wing matching the *allelic dominance model* (p_a) and used the difference of proportions between the two models ($p_a - p_h$) (Supplementary Figure 2). A difference close to 0 indicated that the *colour hierarchy model* accurately predicts the observed direction of dominance and thus validate colour hierarchy as a reliable mechanism of dominance. A negative

value then indicates that the *allelic dominance model* was a better predictor of heterozygotes phenotype than the *colour hierarchy model*.

Heterozygotes with one of the allele from the ancestral class displayed a negative $p_a - p_h$ difference indicating that *allelic dominance model* is a better descriptor of the phenotype for these heterozygotes. All other heterozygous genotypes display values close to 0, indicating that the *colour hierarchy model* is an accurate predictor of the observed direction of dominance between alleles from the derived class. The $p_a - p_h$ difference was significantly lower in heterozygotes composed of ancestral and derived alleles than in heterozygotes with two derived alleles (Nested ANOVA, N = 472, $F_{1,471} = 48.89$; $P < 0.0001$), confirming the existence of two distinct mechanisms of dominance at the supergene *P*, and their segregation between/within allelic classes.

To visualize the consequences of this transgression on the phenotypes of heterozygotes carrying these alleles, we compared wing colour patterns predicted by the *colour hierarchy model* to those actually observed in the controlled crosses (Supplementary Figure 3). This reveals striking differences in pattern, especially in the amount of melanic patches, showing that many black patches carried by the ancestral alleles were recessive.

SUPPLEMENTARY REFERENCES:

1. Joron, M. *et al.* Chromosomal rearrangements maintain a polymorphic supergene controlling butterfly mimicry. *Nature* **477,** 203–206 (2011).
2. Joron, M. *et al.* A Conserved Supergene Locus Controls Colour Pattern Diversity in Heliconius Butterflies. *PLoS Biol.* **4,** e303 (2006).
3. Meyer, F. & Beucher, S. Morphological segmentation. *J. Vis. Commun. Image Represent.* **1,** 21–46 (1990).
4. Meyer, F. Color image segmentation. in *Image Processing and its Applications, 1992., International Conference on* 303–306 (1992).
5. Cheng, H.-D. & Sun, Y. A hierarchical approach to color image segmentation using homogeneity. *Image Process. IEEE Trans. On* **9,** 2071–2082 (2000).
6. Kurugollu, F., Sankur, B. & Harmanci, A. E. Color image segmentation using histogram multithresholding and fusion. *Image Vis. Comput.* **19,** 915–928 (2001).
7. Nikolaev, D. P. & Nikolayev, P. P. Linear color segmentation and its implementation. *Comput. Vis. Image Underst.* **94,** 115–139 (2004).
8. Shafarenko, L., Petrou, H. & Kittler, J. Histogram-based segmentation in a perceptually uniform color space. *Image Process. IEEE Trans. On* **7,** 1354–1358 (1998).
9. Mattes, D., Haynor, D. R., Vesselle, H., Lewellen, T. K. & Eubank, W. Nonrigid multimodality image registration. *Med. Imaging* **4322,** 1609–1620 (2001).
10. Mattes, D., Haynor, D. R., Vesselle, H., Lewellen, T. K. & Eubank, W. PET-CT image registration in the chest using free-form deformations. *Med. Imaging IEEE Trans. On* **22,** 120–128 (2003).
11. Thévenaz, P. & Unser, M. Optimization of mutual information for multiresolution image registration. *Image Process. IEEE Trans. On* **9,** 2083–2099 (2000).
12. Styner, M. & Gerig, G. Evaluation of 2D/3D bias correction with 1+ 1ES-optimization. *Rapp. Rech.* **179,** (1997).
13. Styner, M., Brechbuhler, C., Szckely, G. & Gerig, G. Parametric estimate of intensity inhomogeneities applied to MRI. *Med. Imaging IEEE Trans. On* **19,** 153–165 (2000).

14. Ibanez, L. & Insight Software Consortium. *The ITK software guide*. (Kitware, 2003).

15. Yoo, T. S. *et al.* Engineering and algorithm design for an image processing api: a technical report on itk-the insight toolkit. *Stud. Health Technol. Inform.* 586–592 (2002).

16. Ibanez, L., Schroeder, W., Ng, L. & Cates, J. *The ITK Software Guide Second Edition, 2005*. (2005).

17. Martin, K. & Hoffman, B. *Mastering CMake: a cross-platform build system*. (Kitware Inc., 2003).

18. Rohlf, F. J. & Slice, D. Extensions of the Procrustes Method for the Optimal Superimposition of Landmarks. *Syst. Zool.* **39,** 40–59 (1990).

19. Turk, M. & Pentland, A. Eigenfaces for recognition. *J. Cogn. Neurosci.* **3,** 71–86 (1991).
